# Analysis of MicroRNA Expression Changes During the Course of Therapy In Rectal Cancer Patients

**DOI:** 10.3389/fonc.2021.702258

**Published:** 2021-09-02

**Authors:** Klara Cervena, Vendula Novosadova, Barbara Pardini, Alessio Naccarati, Alena Opattova, Josef Horak, Sona Vodenkova, Tomas Buchler, Pavel Skrobanek, Miroslav Levy, Pavel Vodicka, Veronika Vymetalkova

**Affiliations:** ^1^Department of Molecular Biology of Cancer, Institute of Experimental Medicine of the Czech Academy of Sciences, Prague, Czechia; ^2^Institute of Biology and Medical Genetics, 1^st^Medical Faculty, Charles University, Prague, Czechia; ^3^Czech Centre for Phenogenomics, Institute of Molecular Genetics of the Czech Academy of Sciences, Vestec, Prague, Czechia; ^4^Molecular Genetics Epidemiology Unit, Italian Institute for Genomic Medicine, c/o IRCCS Candiolo,, Turin, Italy; ^5^Candiolo Cancer Institute, FPO-IRCCS, Candiolo, Italy; ^6^Biomedical Centre, Faculty of Medicine in Pilsen, Charles University, Pilsen, Czechia; ^7^Third Faculty of Medicine, Charles University, Prague, Czechia; ^8^Department of Oncology, First Faculty of Medicine, Charles University and Thomayer Hospital, Prague, Czechia; ^9^Department of Surgery, First Faculty of Medicine, Charles University and Thomayer Hospital, Prague, Czechia

**Keywords:** biomarker, microRNA, liquid biopsy, plasma, miR-122-5p, miR-142-5p, rectal cancer

## Abstract

MicroRNAs (miRNAs) regulate gene expression in a tissue-specific manner. However, little is known about the miRNA expression changes induced by the therapy in rectal cancer (RC) patients. We evaluated miRNA expression levels before and after therapy and identified specific miRNA signatures reflecting disease course and treatment responses of RC patients. First, miRNA expression levels were assessed by next-generation sequencing in two plasma samplings (at the time of diagnosis and a year after) from 20 RC patients. MiR-122-5p and miR-142-5p were classified for subsequent validation in plasma and plasma extracellular vesicles (EVs) on an independent group of RC patients (n=107). Due to the intrinsic high differences in miRNA expression levels between samplings, cancer-free individuals (n=51) were included in the validation phase to determine the baseline expression levels of the selected miRNAs. Expression levels of these miRNAs were significantly different between RC patients and controls (for all p <0.001). A year after diagnosis, miRNA expression profiles were significantly modified in patients responding to treatment and were no longer different from those measured in cancer-free individuals. On the other hand, patients not responding to therapy maintained low expression levels in their second sampling (miR-122-5p: plasma: p=0.05, EVs: p=0.007; miR-142-5p: plasma: p=0.008). Besides, overexpression of miR-122-5p and miR-142-5p in RC cell lines inhibited cell growth and survival. This study provides novel evidence that circulating miR-122-5p and miR-142-5p have a high potential for RC screening and early detection as well as for the assessment of patients’ outcomes and the effectiveness of treatment schedule.

## Introduction

MicroRNAs (miRNAs), a class of non-coding RNAs, can modulate gene expression post-transcriptionally by RNA interference (1). Many studies showed that miRNAs target tumor suppressor genes or oncogenes reflect the corresponding effect on tumorigenesis (2–6). Aberrant miRNA expression profiles were identified in tumor patients, but also were identified as biomarkers of cancer prognosis, and/or therapy response (7–9). Several studies have identified miRNAs associated with chemoresistance or tumor chemo- and radiosensitivity (10–12). Most of the studies examined miRNA role as predictors for therapy outcome by comparing their expression between responders and non-responders focusing only on samples collecting either before or only after treatment in colorectal cancer patients (6). Other studies investigated the changes in the expression levels of circulating miRNAs in incident colorectal cancer (13–15). Besides this, little is known about the changes in miRNA expression profiles before and after therapy and if this diversity is associated with the patient’s prognosis. Accumulating evidence about the presence of miRNAs in body fluids has suggested their potential as promising biomarkers with clinical utility (16, 17). Despite modern surgical techniques and improvement of systematic therapy, colorectal cancer still represents the third most common cancer worldwide and one of the leading causes of cancer-related mortality (18). Colorectal cancer is a heterogeneous disease, with colon and rectum having embryologic, anatomical, molecular, therapeutic, and prognostic differences. For these reasons, the two sites should be considered as separate diseases (19–22).

Rectal cancer (RC) accounts approximately for 30% of all colorectal cancer. Unlike the tumor in colon, RC tends to be diagnosed at a younger age, it is more frequently associated with lung-only metastasis and requires a different treatment strategy (23–25). Although neoadjuvant chemoradiotherapy based on 5-fluorouracil (5-FU), followed by surgery and potential adjuvant chemotherapy, represents the standard therapy a significant proportion of RC cases do not profit from this treatment schedule. There is a continuous effort to identify new biomarkers that can predict a patient´s prognosis or improve an earlier RC diagnosis. Liquid biopsy is currently at the center of this research interest.

The present study aimed to describe the changes in miRNA expression in plasma of RC patients before and after therapy and to explain why patients with the same cancer stage may have different treatment susceptibility and long-term outcomes. The expression profiles of selected miRNAs were also compared to those of cancer-free individuals. Identified prognostic miRNAs were further investigated for their involvement in cellular processes (such as proliferation, survival, and impact on cell cycle) in *in vitro* experiments using RC cell lines together with the administration of 5-FU.

## Material and Methods

### Design of the Study

High throughput sequencing of the whole miRNome was performed on plasma samples from 20 patients with RC (Discovery cohort). Specifically, plasma was sampled at two different time-points: at the time of diagnosis (before surgery) and a year after the diagnosis (corresponding to the time of termination of the therapy). Those miRNAs showing the highest differences in expression levels (based on p-value and fold change) between these two samplings, as well as those associated with the therapy response were selected for the validation phase.

Significant miRNA expression levels were further validated by real-time quantitative polymerase chain reaction (RT-qPCR) in plasma of an independent group of RC patients (n=107, Validation cohort) whose sampling was performed in a similar way as the Discovery cohort. Due to the observed high differences in miRNA expression levels between the two samplings, the selected miRNAs were also investigated in a control group consisting of cancer-free individuals (n=51). In addition, for the Validation cohort the expression levels of the selected miRNAs were analyzed also in plasma extracellular vesicles (EVs) in both 2 samplings. For detailed clinical characteristics of patients from both Discovery and Validation cohorts, see **Table 1**.

**Table 1:** Patient’s clinical characteristics.

		Discovery cohort	Validation cohort	Cancer-free individuals
		N (total = 20)	N (total = 107)	N (total = 51)
Age	Years ± SD	62±13	65 ± 11	58 ± 7
Gender	male	13 (65%)	74 (69%)	23
	female	7 (35%)	33 (31%)	28
Stage^a,b^	I	2 (10%)	5 (5 %)	–
	II	8 (40%)	34 (35 %)	
	III	9 (45%)	51 (52 %)	
	IV	1 (5%)	8 (8 %)	
Grade^a^	1	2 (10%)	7 (7%)	–
	2	16 (80%)	79 (77%)	
	3	2 (10%)	16 (16%)	
Survival status^a^	dead	4 (20%)	14 (15%)	–
	alive	16 (80%)	79 (85%)	
Presence of a	Yes	1 (5%)	12 (13%)	–
local recurrence^a^	No	19 (95%)	80 (87%)	
Surgery	Yes	20 (100%)	105 (98%)	–
	No	0 (0%)	2 (2%)	
Neoadjuvant	Yes	10 (50%)	46 (62%)	–
therapy^a^	No	10 (50%)	28 (38%)	
Adjuvant therapy^a^	Yes	11 (61%)	42 (57%)	–
	No	7 (39%)	32 (43%)	

a Numbers may not add up to 100% of available subjects because of missing data.

b Staging was based on pathological staging according to the tumor–node–metastasis (TNM) system Union for International Cancer Control (UICC).

Finally, to partially address their mechanism of action, the selected miRNAs associated with the therapy response were further studied *in vitro* for their effect on cell proliferation, survival, and cell cycle after the administration of 5-FU.

**Figure 1:**
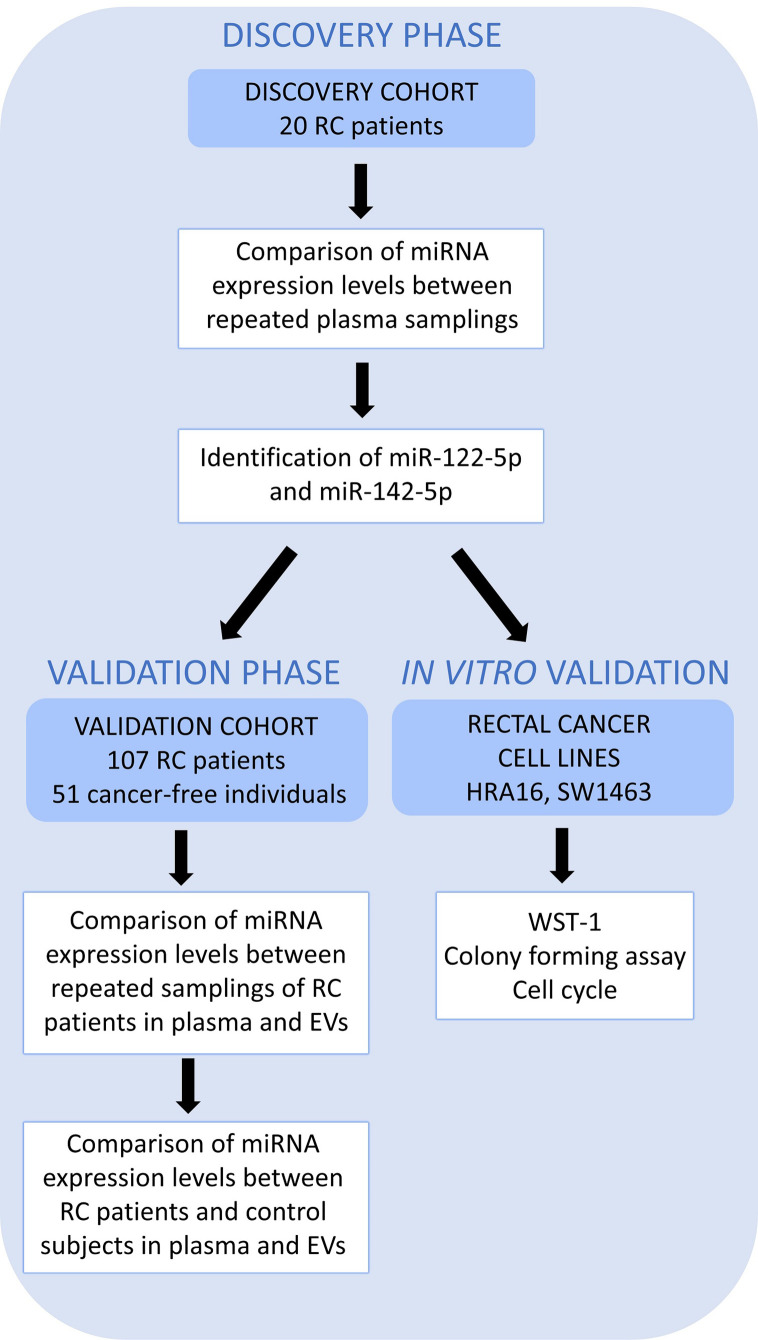
A schematic overview of the workflow. High throughput sequencing (Discovery cohort – 20 RC patients) was performed on whole plasma collected at 2 sampling occasions to identify those miRNAs differentially expressed between two samplings (the first, at the time of diagnosis, T0 and the second, a year after the diagnosis, T1). miR-122-5p and miR-142-5p were validated in plasma and further in plasma EVs on an independent group of RC patients (n=107, Validation cohort) at the same 2 sampling occasions. Both miRNAs were also investigated in a control group consisting of cancer-free individuals (n=51) by RT-qPCR. The effect on cell proliferation, survival, and cell cycle was also studied on rectal cancer cell lines (*in vitro studies*).

A simplified workflow of the study is depicted in **Figure 1**.

### Study Population and Collection of Biological Specimens

The Discovery cohort comprised of 20 patients with RC while the Validation cohort consisted of 107 RC patients and 51 cancer-free volunteers. All patients were recruited between 2007 and 2018 at the Department of Surgery, the Thomayer Hospital in Prague, Czech Republic. Study participants provided information on their lifestyle, body mass index (BMI), and family/personal history of cancer, using a structured questionnaire to determine basic demographic characteristics and potential risk factors for RC. All patients were followed up until August 2020. Clinical data of all patients are represented by clinical stage at diagnosis (classified as the Tumor-Node-Metastasis (TNM) system according to Union for International Cancer Control), grade, information about neoadjuvant and adjuvant treatment regimens, the presence of recurrence, and survival. In both the Discovery and Validation cohorts, RC patients were divided into responders (those who benefit from the chemotherapy and with no relapses) and non-responders (patients with lack of any therapy response, or those that died very early after diagnosis).

For all patients and controls, peripheral blood was collected into EDTA tubes, stored at 4°C, and centrifugated at 1400 rpm at 4°C for 10 min within 1 hour after its collection for plasma separation. The plasma fraction was immediately frozen and preserved at -80°C.

All study individuals signed a written consent to participate in the study and approved the use of their biological samples for genetic analyses according to the Helsinki declaration. The design of the study was approved by the Ethics Committee of the Institute of Experimental Medicine and the Thomayer Hospital, Prague, Czech Republic.

### MiRNA Isolation From Whole Plasma and Plasma EVs

#### RNA Extraction and Quality Control

RNA from whole plasma was extracted using Plasma/Serum Circulating and Exosomal RNA Purification Kit (Norgen Biotek, Canada) according to manufacturer´s protocol.

ExoQuick exosome precipitation solution (System Biosciences, USA) was used for EVs precipitation from plasma according to manufacturers’ instructions and as described by us (26). Briefly, 200 μl plasma was mixed with 50.4 μl of ExoQuick solution and refrigerated at 4°C overnight (at least 12h). The mixture was centrifuged at 1500xg at 4°C for 30min. The EVs pellet was dissolved in 200 μl of nuclease-free water and RNA was extracted immediately from the solution using Plasma/Serum Circulating and Exosomal RNA Purification Kit (Norgen Biotek, Canada).

For all samples, RNA concentration was quantified with Qubit 3.0 Fluorometer using the Qubit microRNA assay Kit (Thermo Fisher Scientific, USA).

#### Small RNA-Sequencing

The next generation sequencing library preparation for the Discovery set was carried out as described in Sabo et al. (26). MiRNA libraries were constructed using the NEB Next Multiplex Small RNA Library Prep Set for Illumina (New England BioLabs Inc., USA) according to the manufacturer’s protocols. Briefly, miRNA samples (5-10 ng) were ligated with 5´and 3´adapters, followed by reverse transcription- for complementary DNA (cDNA) library construction and incorporation of index tags. The cDNA library fragments were purified by AMP Pure XP Beads and separated on a 6% TBE PAGE gel and 145–160 bp size fraction containing miRNA inserts was isolated. The twenty cDNA library samples were pooled in equimolar amounts and used for cluster generation and sequence analysis in a single lane on an Illumina HiSeq2000 (50 bp single read). This work was performed at the Italian Institute for Genomic Medicine.

Sequencing data analysis was conducted using the miARma-Seq pipeline (http://miarmaseq.cbbio.es/). miARma-Seq is a new comprehensive pipeline analysis suite designed for mRNA, miRNA, and circRNA identification and differential expression analysis, applicable to any sequenced organism. It integrates, among others, the Bowtie tool for read mapping and the miRDeep2 tool for miRNA expression analysis and *de novo* miRNA prediction (27). Quality control was performed using FastQC (version 0.11.5). The results for individual samples were merged into the summary report using MultiQC. Read length was constant for all samples (51 bp). Data were then filtered using Trimmomatic tool (version 0.36), with the following parameters: i) SLIDINGWINDOW:4:20 - trimming of low-quality bases from 3’ end using sliding window; ii) TRAILING:20 - trimming of low-quality bases from 3’ (with base quality <20); iii) ILLUMINACLIP: Adapter_sequences. fa: 4:20:5 - trimming of adapter sequences; vi) MINLEN:15 - discarding reads of length > 15 bp. After filtering, read length peaked around 22 and 32 bp, respectively.

All the samples were mapped to the i) reference human genome (hg38 release), ii) known human matured miRNAs (using the miRanalyzer web server tool (http://bioinfo2.ugr.es/miRanalyzer/miRanalyzer.php), iii) known human miRNA hairpins (miRBase database, version 22), and vi) human tRNAs (UCSC database). The alignment to the human tRNA sequences was performed to reveal potential contaminations.

### MiRNA Expression Analysis by RT-qPCR

For evaluating miRNA expression levels in whole plasma and plasma EVs in the Validation cohort, cDNA was synthesized from total RNA by TaqMan MicroRNA Reverse Transcription Kit (Applied Biosystems, USA) and analyzed using TaqMan MicroRNA assays (hsa-miR-142-5p - ID 002248, hsa-miR-122-5p – ID 002245, *RNU48* – ID 001006, *RNU6B* – ID 001093, Applied Biosystems, USA) according to the manufacturer´s protocol. *RNU48* and *RNU6B* were used as reference genes selected by Normfinder (GenEx Enterprise, MultiD, Thermo Fisher Scientific). For 10 μl reverse transcription reaction, 5 μl of RNA sample (40 ng RNA) were used to prepare cDNA and reaction mixtures were incubated for 30 min at 16°C, 30 min at 42°C, 5 min at 85°C and then held at 4°C (MJ Research PTC-200 Thermal Cycler, Marshall Scientific).

All samples were pre-amplified prior to use in RT-qPCR using an IQ SuperMix (Bio-Rad, USA). The pre-amplification reaction contained 2 μl cDNA, 1.5 μl miRNA primer, 5 μl IQ SuperMix and 1.5 μl RNAse free water. Preamplification was performed as follows: 95°C for 3 min, 18 cycles of 95°C for 15 sec and 59°C for 4 min and then held at 4°C (MJ Research PTC-200 Thermal Cycler, Marshall Scientific). After pre-amplification, each reaction mixture was diluted 100x.

RT-qPCR was performed using the Applied Biosystems 7500 Sequence Detection System. The 20 μl PCR reaction mixture consisted of 2 μl of 100x diluted pre-amplified cDNA, 1 μl of primer (TaqMan microRNA Assay kit, Applied Biosystems, USA), 10 μl TaqMan Universal MasterMix II – no UNG (Applied Biosystems, USA) and 7 μl RNAse-free water. Reactions were run in a 96-well optical plate at 95°C for 10 min, followed by 40 cycles at 95°C for 15 sec and 60°C for 10 min.

### Cell Lines and Cell Culture

Both RC cell lines (HRA16 and SW1463) were originally obtained from ECACC (Sigma Aldrich, USA). The cells were confirmed free from mycoplasma contamination with MycoAlert PLUS Mycoplasma Detection Kit (Lonza, Switzerland). HRA16 and SW1463 were cultured in a humidified atmosphere at 37°C and 5% CO_2_ in Dulbecco´s Modified Eagle Medium (DMEM, Sigma Aldrich, USA) and in Roswell Park Memorial Institute (RPMI-1640, Sigma Aldrich, USA), respectively, and supplemented with 10% fetal bovine serum (FBS, Gibco, Thermo Fisher Scientific), 1 mM L-Glutamine, 100 U/ml penicillin/streptomycin and, in case of RPMI, 1 mM sodium pyruvate (Biosera). Cells were used up to 8 passages.

### Cell Transfection and Treatment

Cells were transfected 24 hours after seeding with 10 nM hsa-miR-142-5p (HMI0068; Sigma Aldrich, USA) and hsa-miR-122-5p (HMI0218; Sigma Aldrich, USA) mimics and miRNA mimics negative control with no homology to the human genome (HMC0003; Sigma Aldrich, USA) using Lipofectamine^®^ RNAiMAX (Invitrogen, USA) according to the manufacturer’s protocol. All the experiments were performed in three independent replicates. The transfection efficiency was confirmed by RT-qPCR. After transfection, 5-FU (Sigma Aldrich, USA) was dissolved in DMSO (Sigma Aldrich, USA) to 5 µM 5-FU, added to selected wells, and cells were treated for 24 hours.

### Cell Proliferation (WST-1 Assay) and Colony Forming Assay

The proliferative capacity in miRNA-modulated cells (750 000 cells/ml) was measured using a water-soluble tetrazolium-1 solution (WST-1 proliferation assay, Roche, USA) 48 hours after transfection. The WST-1 reagent was added into the media according to the manufacturer´s instructions and measured at 450 and 690 nm. Absorbance was measured using fluorescence reader Biotek ELx808 (Biotek, USA) and proliferation was monitored for different time points (24, 48, 72 hours). For colony forming assay, transfected cells were plated on 6-well plates (500 cells per well). After 24 hours, cells were treated with 5 μM 5-FU for 24 hours, and then the medium was replaced with fresh medium. After 12 days, colonies were fixed with 3% formaldehyde and stained with 1% crystal violet. The number of colonies was counted manually. Both measurements were performed in triplicates.

### Cell Cycle Analysis

Cells (750 000 cells/ml) were seeded on 12-well plates (with and without 5-FU treatment), harvested by trypsinization, washed with PBS, and spun down at 1000 rpm at room temperature for 10 min. Then, 1 ml of Propidium iodide (PI) staining solution (0.02 mg/ml PI, 0.02 mg/ml RNase, 0.05% Triton X-100) was added to the cell pellet and cells were incubated for 30 min at 37°C in the dark. After incubation, samples were measured using flow cytometer Apogee A-50 micro (Apogee, USA). Measured data were analyzed with Flowlogic software (Inivai Technologies, Australia).

### External Validation Through TCGA Data

All miRNA-Seq transcriptional profiles and detailed clinical information were downloaded from The Cancer Genome Atlas TCGA (https://portal.gdc.cancer.gov) using the TCGA biolinks R package (28). For the present study, data from the project TCGA-READ (rectal adenocarcinoma, n=155) were analysed and filtered according to the following criteria: 1) analyses were performed on rectal tumor patients only (ICD-10 Classification code “C20”) which have miR-122 and miR-142 expression levels data available and 2) clinical data including survival data should also be available. Finally, a total of 74 RC patients presented expression levels of miR-142 and 22 RC patients had data for miR-122.

### Statistical Analysis

Differential expression of the miRNA-Seq raw count data was assessed using the EdgeR and Noiseq directly using the miARma-Seq pipeline. Due to the small sample size and the heterogeneity of the RC phenotype, unadjusted p-values were used. Statistical significance was set up at p ≤ 0.05. RT-qPCR statistical analysis was performed by GenEx qPCR data analysis software version 6 (multiD) and R version 3.4.0. The linear mixed models were used (R package lmer and multcomp) for the comparison of miRNA expression in different conditions and different tissues. *In vitro* analyses were evaluated using the Mann-Whitney test in GraphPad Prism 8 (GraphPad Software, San Diego, CA, USA). Statistical analysis for TCGA data was performed using the R environment using the dplyr and survival, survminer and ggplot2 package. The survival significance was measured by log-rank test and Mann-Whitney was employed for the differential expression analyses. The significance was set to non-adjusted p = 0.05. The multivariate Cox regression analysis was performed in the R environment using the olsrr package and the ols_step_all_possible function that uses the Akaike information criterion (AIC) criterion for all possible combinations of factors on the patient dataset after screening and EVs and plasma specimen.

### Bioinformatics Analysis

#### Identification of miRNAs as Potential Pharmacogenomic Biomarkers for Anticancer Drugs

To identify potential pharmacogenomic biomarkers characterized by miRNA expression and discover the underlying mechanisms of anticancer drug responses mediated by miRNAs, the Small Molecule-miRNA Network-Based Inference [SMiR-NBI; http://lmmd.ecust.edu.cn/database/smir-nbi/ (29–31)] model was used. The SMiR-NBI model was built on the basis of a heterogeneous network connecting drugs, miRNAs, and genes [34] and predicts interactions between small molecules and miRNAs.

#### Identification of Validated Targets for Studied MiRNAs

The miRWalk tool was used to conduct *in silico* prediction of miRNA targets (miRNA-mRNA Interaction Analysis). miRWalk, an open-source platform, generates up-to-date predicted and validated miRNA-binding sites of known genes [http://mirwalk.umm.uni-heidelberg.de (32)]. The gene set enrichment analysis (GSEA) was employed to test whether any functional group of genes (e.g. pathways, the target of a transcription factor) from the list of target genes were significantly enriched in specific pathways or molecular functions.

## Results

### Discovery Cohort

In the 20 RC patients forming the Discovery cohort, sixty-three miRNAs resulted significantly differently expressed between the two plasma samplings. MiR-122-5p showed the highest difference between consecutive samplings and was selected for further validation. With the advantage of long-term follow-up, we additionally evaluated the differentially expressed miRNAs between consecutive samplings and correlated the results with the response to therapy; from this last evaluation we identified also miR-142-5p for further validation.

miR-122-5p expression levels were significantly higher in the second sampling than in the first sampling (p<0.001, 2.9-fold change). miR-142-5p was identified as associated with patients’ therapy response: non-responders (i.e. patients who died within a year after the diagnosis, did not benefit from therapy, or had a local recurrence) exhibited significantly lower expression levels in their second sampling compared to the first one (p<0.001, -10.3-fold change). Downregulation of these miRNAs was calculated comparing the second sampling *vs* the first sampling.

### Validation by RT-qPCR

**Figure 2:**
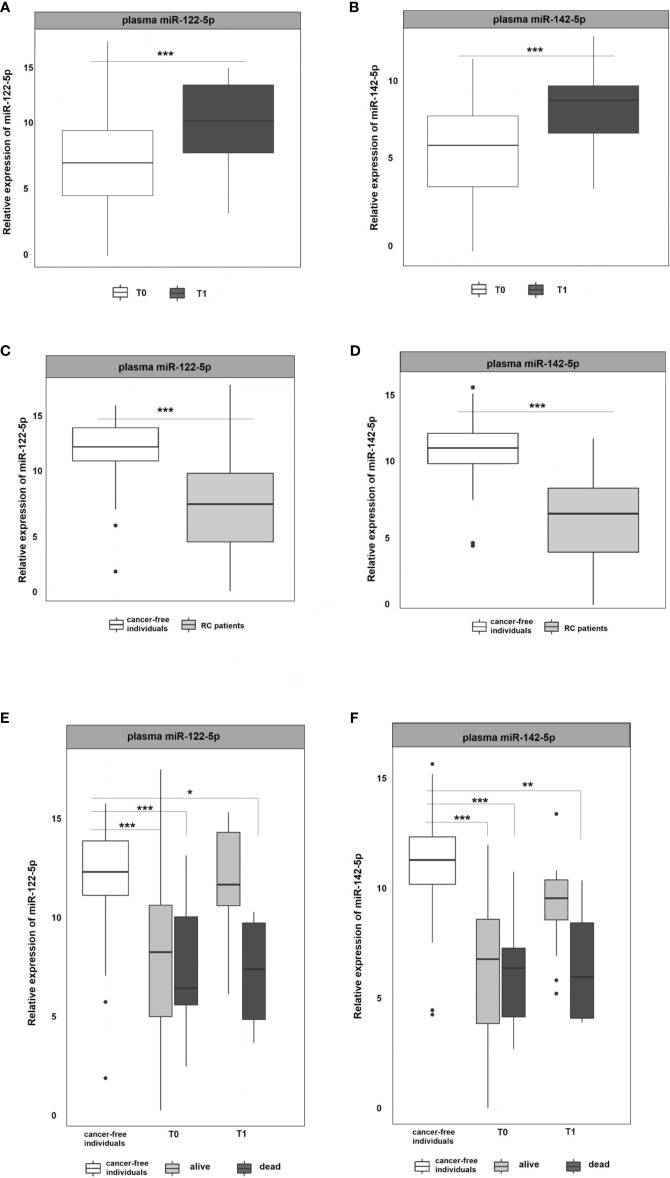
Expression analysis of miR-122-5p and miR-142-5p in plasma. **(A, B)** The expression levels of miR-122-5p and miR-142-5p were significantly lower at the 1^st^ plasma sampling (T0) compared to 2^nd^ sampling (T1) of RC patients (miR-122-5p: p=0.0003, 3.2-fold change; miR-142-5p: p=0.0002, 2.4-fold change). **(C, D)** Expression levels of miR-122-5p and miR-142-5p were significantly lower in plasma from RC patients compared to the plasma of cancer-free individuals (for all p <0.001). **(E, F)** A year from the diagnosis (2^nd^ sampling, T1), a significant difference was observed between patients that do not respond a year after diagnosis and cancer-free individuals (miR-122-5p: plasma: 4.9-fold change, p=0.05; miR-142-5p: plasma: 4.5-fold change, p=0.008). *p ≤ 0.05, **p ≤ 0.01, ***p ≤ 0.001.

**Figure 3:**
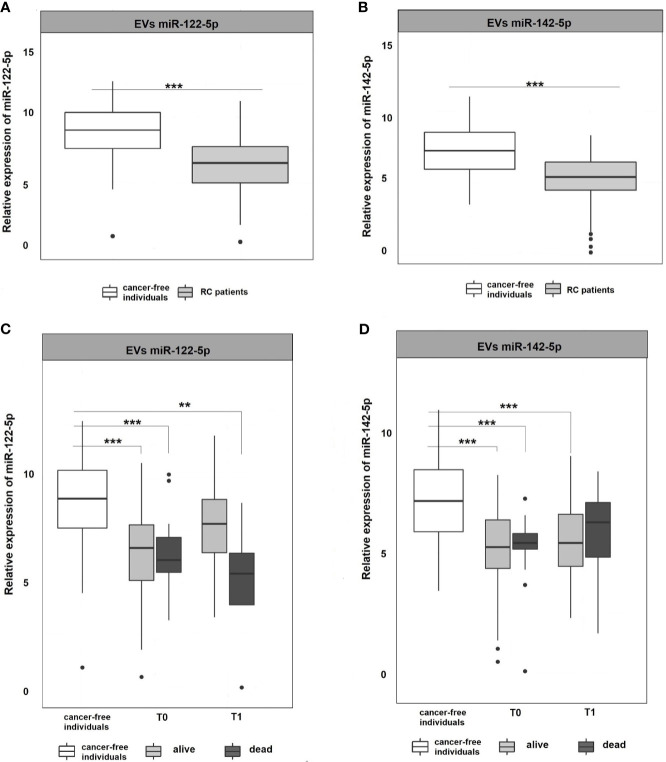
Expression analysis of miR-122-5p and miR-142-5p in EVs. **(A, B)** Expression levels of miR-122-5p and miR-142-5p were significantly lower in plasma EVs of RC patients compared to EVs of cancer-free individuals (for all p <0.001). **(C, D)** A year from the diagnosis (2^nd^ sampling, T1), a significant difference was observed between patients that do not respond a year after diagnosis and cancer-free individuals for miR-122-5p (4.9-fold change, p=0.007). This trend was not observed for miR-142-5p. *p ≤ 0.05, **p ≤ 0.01, ***p ≤ 0.001.

miR-122-5p and miR-142-5p were validated by RT-qPCR in whole plasma and further in plasma EVs of RC patients (n=107) and cancer-free controls (n=51). Both analyzed miRNAs were detectable in whole plasma and plasma EVs. Results are represented in **Figures 2** and **3**.

As in the Discovery cohort, in the Validation cohort miR-122-5p was significantly upregulated in the second (T1) plasma sampling when compared with the first one (T0) of RC patients. miR-122-5p expression levels in second sampling (T1) were significantly higher than those in the first sampling (T0; p=0.0003, 3.2-fold change**;** **Figure 2A**). Interestingly, the same effect was observed for miR-142-5p that was associated with the response to therapy in the Discovery cohort (p=0.0002, 2.4-fold change; **Figure 2B**).

Due to the observed differences between consecutive plasma samplings in RC patients, we wanted to determine the baseline expression levels of selected miRNAs. For this reason, we included plasma samples from a control group consisting of healthy volunteers and compared the miRNA expression levels of RC patients at the first sampling (T0 at the time of the diagnosis) with those from the control group. The results revealed that both, miR-122-5p and miR-142-5p, were significantly down-regulated in RC patients when compared with the control group in plasma samples (for all the analyses p <0.001; miR-122-5p: -4.5-fold change, miR-142-5p:-4.8-fold change; **Figures 2C, D** **)**.

With the advantage of long-term follow-up and gathering the clinical data, we further stratified the patients according to the response to therapy. Identified miRNAs were further evaluated for patient´s clinical characteristics and treatment response (recurrence of the tumor after the therapy and 3-year survival of the patients since diagnosis). At the time of the diagnosis (1^st^ sampling, T0), the expression levels of both tested miRNAs did not differ between patients that benefited from the therapy and were alive from those dying during the follow-up period (**Figures 2E, F**). A year after the diagnosis (2^nd^ sampling, T1), both miRNA expression profiles were significantly modified in responding patients and no longer different from those measured in controls (**Figures 2E, F** **)**. On the contrary, we observed a significant difference between patients that do not respond a year after diagnosis and the control group (miR-122-5p: plasma: 4.9-fold change, p=0.05; miR-142-5p: plasma: 4.5-fold change, p=0.008, **Figures 2E, F** **)**.

EVs are cell-derived membranous structures of endocytic origin and contains various functional protein, mRNA, or miRNAs (33). Despite their promising potential, the clinical use is still limited. Today, several studies focused on comparing plasma and EVs miRNAs as non-invasive biomarkers (34, 35). Following these evidences, miRNA expression levels were also analyzed in plasma EVs to see if their expression profiles in EVs mimics those in plasma. The results revealed that both miR-122-5p and miR-142-5p in plasma EVs were significantly down-regulated in RC patients at the time of the diagnosis (T0) when compared with cancer-free controls (for all analyses, p <0.001; miR-122-5p: -2.3-fold change, miR-142-5p -2.1-fold change; **Figures 3A, B** **)**. We did not observe any difference between consecutive plasma EVs samplings. However, after stratification of RC patients based on therapy response, we observed a similar trend as in plasma of RC patients (data not shown). Concerning miR-122-5p EVs expression levels a year after diagnosis (T1), the expression levels in RC patients that benefited from therapy were not different from the expression levels in the control group while patients that did not respond to therapy still evinced lower miR-122-5p expression levels (comparison with controls: miR-122-5p: 4.9-fold change, p=0.007, **Figures 3C, D** **)**. This trend was not observed for miR-142-5p in plasma EVs (1.6-fold change, p=0.4). This discrepancy might be caused by a relatively small number of patients that evinced relapse and did not respond well to therapy.

No significant differences were observed between different stages of RC, neither for miR-122-5p nor for miR-142-5p in EVs and plasma. These non-significant outcomes can be attributed to the small number of patients in some of the stages (I=5, II=34, III=51, and IV=8, **Supplementary Figure 1**).

### Cell-Culture-Based Assessment

#### The Impact of miRNAs on Proliferation, Colony Formation, and Cell Cycle Distribution

To prove the effect of miR-122-5p ad miR-142-5p on RC pathogenesis and prognosis, RC cell lines SW1463 and HRA16 were further analyzed. The over-expression of both miRNAs was tested in the RC cells and measured for their ability to affect the proliferation, to form single-cell colonies, and for their impact on the cell cycle. The significant increase in the expression levels of both analyzed miRNAs following the miRNA mimics transfection was confirmed by RT-qPCR.

**Figure 4:**
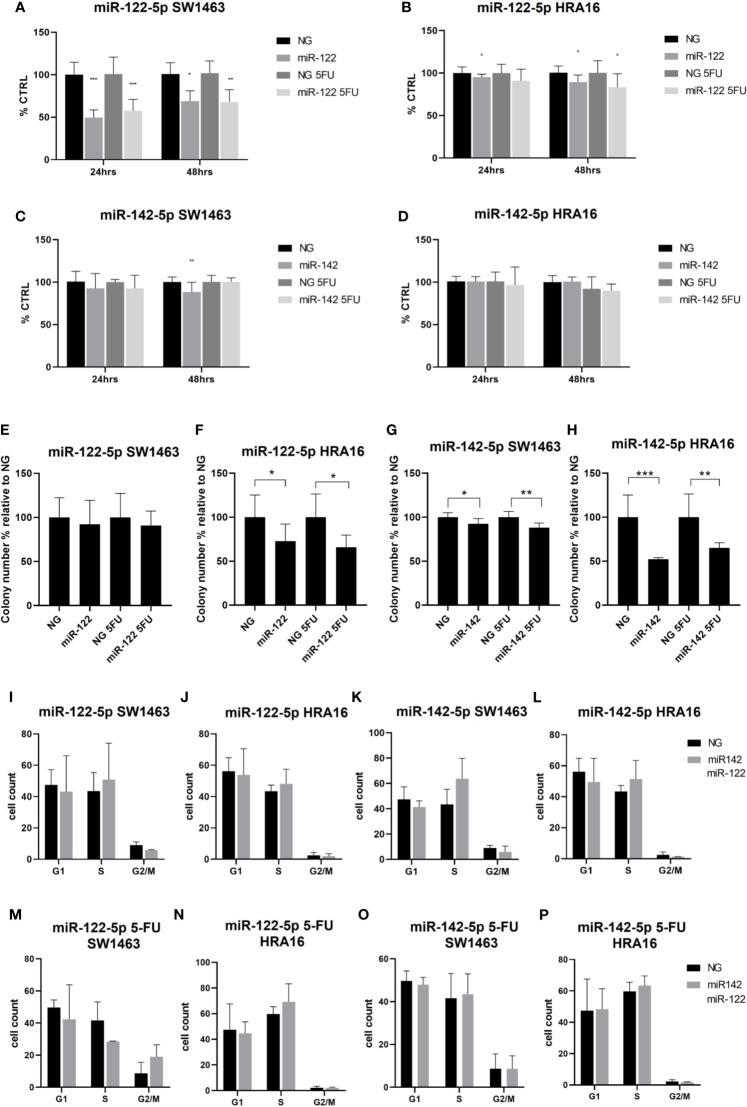
Cell-culture-based assessment. The effect of miR-122-5p and miR-142-5p on cell proliferation **(A–D)**, colony-forming activity **(E–H)**, and cell cycle distribution **(I-P)** in rectal cancer cell lines. **(A, B)** - A significant decrease in cell proliferation of SW1463 cells after the miR-122-5p transfection **(A)** was observed after both 24h and 48h (24h: without 5-FU: p<0.001, with 5-FU: p=0.0003; 48h: without 5-FU: p=0.2, with 5-FU: p=0.008). Same trend was recorded for HRA16 [**(B)**, for 24 h: p=0.05, for 48 h: p= 0.04]. **(C, D)** miR-142-5p transfection had a moderate impact on SW1463 proliferation [**(C)**, p=0.007] but no effect on HRA16 proliferation **(D)**. **(E, F–H)** Both HRA16 and SW1463 transfected with miR-122-5p and miR-142-5p mimics formed significantly fewer colonies than those transfected with control oligonucleotide (miR-122-5p: SW1463 - no significant results, HRA16 – p=0.05; miR-142-5p: SW1463 – p=0.02, HRA16 – p=0.001). The same effect was also observed after the 5-FU administration (miR-122-5p + 5-FU: SW1463 – no significant results, HRA16 – p=0.01; miR-142-5p + 5FU: SW1463 – p=0.0008, HRA16 – p=0.005). **(I–P)** No significant change in cell cycle progression after miR-122-5p and miR-142-5p transfection was observed. *p ≤ 0.05, **p ≤ 0.01, ***p ≤ 0.001.

The effect of miR-122-5p and miR-142-5p (with and without 5-FU) on proliferation activity of SW1463 and HRA16 was measured by WST-1 assay. After 24h, a significant decrease in cell proliferation of SW1463 cells after the miR-122-5p transfection was observed (**Figure 4A**, without 5-FU: p<0.001, with 5-FU: p=0.0003), and the same trend was also recorded after 48 h (**Figure 4A**, without 5-FU: p=0.2, with 5-FU: p=0.008). miR-122-5p also affected the cell proliferation of HRA16 cells (**Figure 4B**, for 24 h: p=0.05, for 48 h: p= 0.04). miR-142-5p had a moderate impact on SW1463 proliferation (**Figure 4C**, p=0.007), whereas no effect on proliferation was recorded in HRA16 transfected cells with miR-142-5p mimics (**Figure 4D**).

To investigate the long-term survival of cancer cells, the colony-forming assay was performed. After 12 days, both cell lines transfected with miRNA mimics formed significantly fewer colonies than those transfected with control oligonucleotide (**Figures 4E–H** and **Supplementary Figure 2**, miR-122-5p: SW1463 - no significant results, HRA16 – p=0.05; miR-142-5p: SW1463 – p=0.02, HRA16 – p=0.001). The same effect was also observed after the 5-FU administration (miR-122-5p + 5-FU: SW1463 – no significant results, HRA16 – p=0.01; miR-142-5p + 5FU: SW1463 – p=0.0008, HRA16 – p=0.005). These results suggest that high levels of both miRNAs, as observed in human samples, might mediate the anti-proliferative effect in tumor cells.

Given these results, we investigated whether these miRNAs have an impact on cell cycle arrest (**Figures 4I–P** and **Supplementary Figure 2**). In both cell lines, we did not observe any significant changes in cell cycle progression after miR-122-5p and miR-142-5p transfection.

### External Validation Through TCGA Data

**Figure 5:**
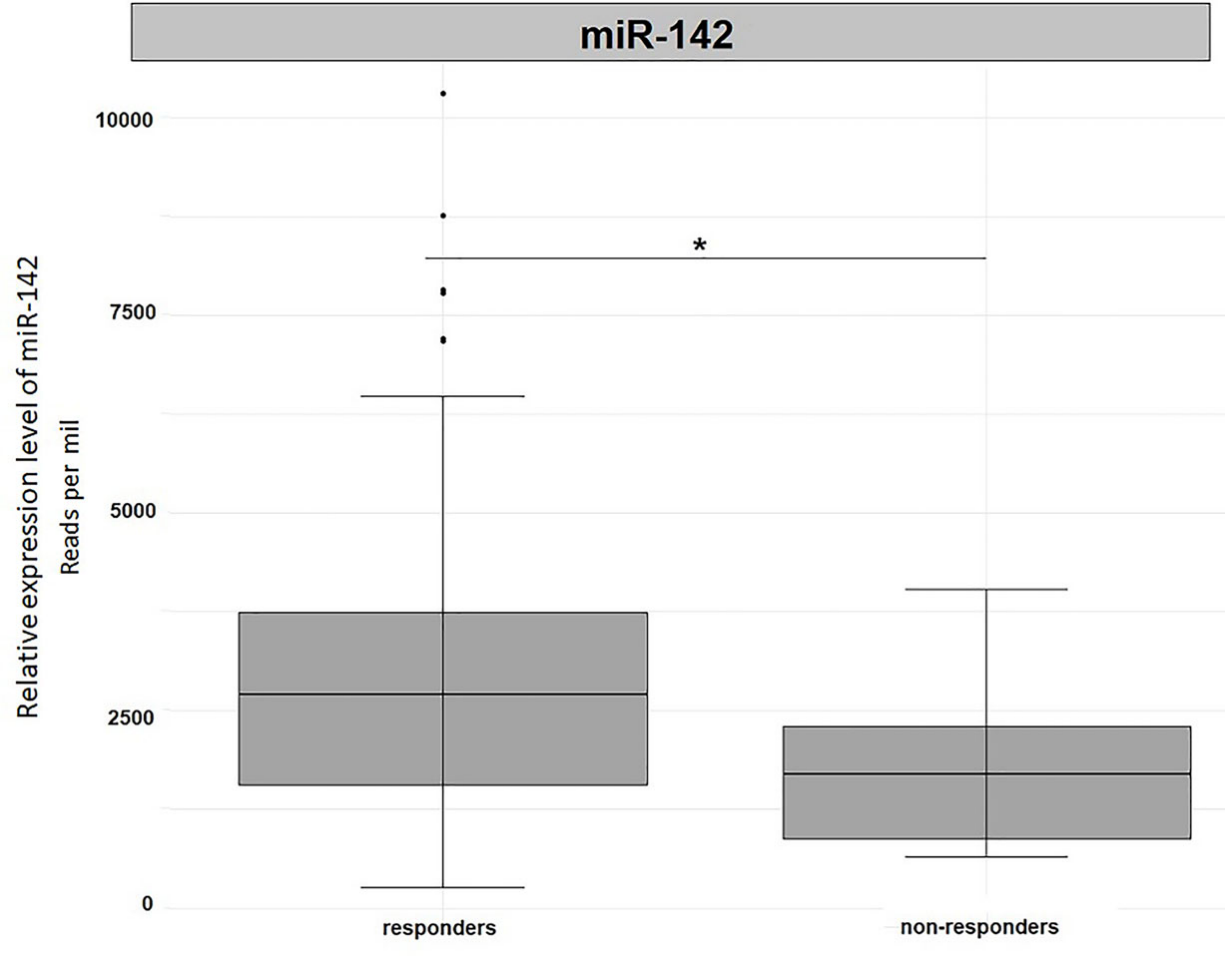
Differential expression analysis of miR-142 in tumor tissues from TCGA-READ project stratified for their responsiveness or not to therapy. *p ≤ 0.05.

In order to validate our results, we used the miRNA expression levels reported in the TCGA project (The Cancer Genome Atlas Rectum Adenocarcinoma TCGA-READ portal). We focused only on those samples included in the TCGA-READ project that had clinical data including survival data, reported miR-122 or miR-142 expression levels. Unfortunately, for miR-122 expression levels were available for 22 patients only and no significant outcomes were noticed (data not shown). Concerning miR-142 expression levels, in non-responders we observed a significantly reduced expression levels for this miRNA when compared to responders (Fold change=1.69, p=0.05, **Figure 5**). This outcome is in agreement with our results.

### Multivariate Analysis of Survival With Clinical Factors and the miRNA Signature

Factors affecting the survival (such as sex, `hsa-miR-142`,` hsamiR-122`, pT, pN, pM, Administration of Neoadjuvant chemotherapy, Administration of Adjuvant chemotherapy) were analyzed using the selection method variables (stepwise selection). This analysis was performed for EVs and plasma samples either considering the expression levels measured in these specimens together or separately.

The overall survival for EVs and plasma samples analyzed together was predominantly dependent on pN, sex, pM and Adjuvant chemotherapy (AIC=67.26).

By ranking the significance of the combinations of individual factors according to the AIC criterion, the combination `hsa-miR-142`-pN-sex-pM-Adjuvant chemotherapy ranked 9th (AIC=69.18), and the combination` hsa-miR-122`-pN-sex-pM-Adjuvant chemotherapy ranked 11th (AIC=69.22) out of a list 511 possible combinations. These results indicated the potential prediction of survival status of both miRNAs.

For the analysis on plasma only, the combination pN and Adjuvant chemotherapy was the strongest predictors (AIC=37.18). Besides, the 8th and 9th place of 255 possible were taken by the combinations containing `hsa-miR-122`-pN-Adjuvant chemotherapy (AIC=39.05) and ` hsa-miR-142`-pN-Adjuvant chemotherapy (AIC=39.16), respectively.

Considering EVs only, the outcomes were similar to those obtained in plasma. The most significant results were for the parameters pN and Adjuvant chemotherapy (AIC=40.89) while the combination `hsa-miR-122`-pN-Adjuvant chemotherapy (AIC=42.09) and` hsa-miR-142`-pN-Adjuvant chemotherapy (AIC=42.85) were on the 3rd and 7th position, respectively.

In summary, the majority of tested models (approx. 70%) included miRNAs as significant predictors (data not shown).

### Bioinformatic Analysis

#### Identification of miRNAs as Potential Pharmacogenomic Biomarkers for Anticancer Drugs

To identify whether both tested miRNAs could work as potential pharmacogenomic biomarkers for anticancer drugs, we have performed an additional analysis with the implementation of SMiR-NBI model which predicts interactions between small molecules and miRNAs (**Table 2**). According to this *in silico* analysis, both miRNAs resulted regulated by many drugs, such as cisplatin or 5-FU. This *in silico* outcome agrees with our findings that both RC cell lines with artificially increased miRNA levels had worse survival after 5-FU therapy.

**Table 2:** The prediction of interaction of small molecules with miR-122-5p and miR-142-5p.

miRNA	Small Molecule	Regulation
hsa-miR-122-5p	Arsenic Trioxide	↑
	1,2,6-Tri-O-galloyl-beta-D-glucopyranose	↓
	Microcystin-LR	↓
	MG132	↑
	Bortezomib	↑
	Cisplatin and 5-Fluorouracil	↑
	Doxorubicin	↑
	Vitamin E	↓
	Enoxacin	↑
	
hsa-miR-142-5p	Triptolide	↓
	Cisplatin	↑
	Progesterone	↑
	Topotecan	↓

↓ miRNA expression was repressed by the listed small molecule.

↑ miRNA expression was upregulated by the listed small molecule.

#### Identification of Validated Targets for Studied miRNAs

Firstly, we identified miRNA targets that have previously been predicted or validated by experimental approaches through miRWalk algorithm and the other tools (including TargetScan, miRTarBase, and miRDB) included in miRWalk. Target genes that could be predicted in at least three databases were defined as highly predicted miRNA targets. For these target genes, Gene Ontology (GO) and Kyoto Encyclopedia of Genes and Genomes (KEGG) pathway analyses were conducted by GSEA.

For miR-122-5p, 236 target genes were predicted according to miRWalk and validated in miRTarBase (**Supplementary Table 1**). Several terms in KEGG, such as “PI3K-Akt signaling pathway”, “Pathways in cancer,” and “Metabolism in cancer” were enriched (**Supplementary Table 2**). After consideration for Benjamini-Hochberg corrections, all these terms were not considered significant. GO analysis of the validated and predicted miRNA targets was conducted, and a total of 6 biological processes (BP), 3 molecular functions (MF), and 3 cellular components (CC) terms were identified. Among them, “Negative regulation of apoptotic process” and “protein phosphorylation” were two of the tops from BP (**Supplementary Table 3**). Interestingly, from Reactome analysis, the neutrophil degranulation showed the only outcome. In the response to infection, neutrophils leave the circulation and migrate towards the inflammatory focus. This result seems to be relevant as inflammation is also likely to be involved in other forms of sporadic as well as heritable colon cancer.

For miRNA-142-5p, 8 target genes predicted in miRWalk were also validated according to miRTarBase (**Supplementary Table 4**). Due to low number of validated target genes, the GSEA analysis was not possible to be performed.

## Discussion

RC disease course and prognosis are heterogeneous and for a proper disease management the discovery of new biomarkers for early diagnosis and treatment response prediction is necessary. In this study, we initially profiled miRNA expression levels of 20 RC patients with consecutive plasma samples by next generation sequencing. Two miRNAs (miR-122-5p and miR-142-5p) were selected for further validation. To investigate whether these miRNAs might be used as non-invasive diagnostic and prognostic biomarkers, miR-122-5p and miR-142-5p were analyzed in plasma and further in plasma EVs of 107 RC patients and 51 cancer-free individuals. Plasma and plasma EVs of RC patients were examined at the time of diagnosis and one year after. EVs are considered as both mediators of cell-to-cell communication, and as potential delivery vesicles for a therapeutic approach (36, 37). We identified miR-122-5p and miR-142-5p as down-regulated in plasma and plasma EVs of RC patients compared to cancer-free individuals. Accordingly, patients with a persistent low miR-122-5p expression levels (in whole plasma and plasma EVs) and miR-142-5p (in whole plasma) in their second sample displayed worse survival. The different expression trends between T1 responders and non-responders in plasma EVs was observed in our study for miR-142-5p. At the time of diagnosis, these differences were not significant. Since we did not observe a similar trend for miR-122-5p, we assumed that the limited number of patients analyzed might be one of the possible explanations. We hypothesize that this observation may be also related to the biological function of miR-142-5p. Sukma Dewi et al. (38) pointed to functional significance of miR-142-3p in establishing cell-to-cell communication. Li and Li (39), observed that miR-142-3p clearly upregulated the cancer stem cells (CSCs) colon population. In their study, authors reported that miR-142-3p in EVs was the main factor for increasing the colon CSC population in colon cancer. The authors also found that this miRNA inhibited the proliferation of colon cancer cells. However, they also showed that miR-142-3p promoted the population of CSCs in colon cancer as a tumour booster. Due to the biological similarity of miRNAs -3p and -5p, we assume the same effect for both isoforms, although this has not yet been described in the literature. We also believe that the same applies also for RC. This may be the reason why in our study, patients who did not respond to treatment had higher levels of miR-142-5p both at the time of diagnosis and one year after diagnosis. However, many questions still remain to be answered about the biological role and mechanism of action of miR-142-5p in RC.

To further explain our observation, both miRNAs were artificially overexpressed in RC cell lines (SW1463 and HRA16) and investigated for their effect on long-term survival with and without administration of 5-FU. Increased levels of both miRNAs in RC cell lines were associated with significantly less formed colonies after administration of 5-FU. These results are in concordance with RC patients and suggest that miR-122-5p and miR-142-5p act as tumor suppressors. In addition, it has been proved that miR-122-5p is up-regulated in the presence of 5-FU by SMiR-NBI model and it might be assumed that the miRNA effect is thus potentiated. Studies connected to colon and rectal pathogenesis and miR-122-5p expression levels were mainly focused on metastatic colorectal cancer. One of the most frequent sites for colon and rectal cancer metastasis is liver and miR-122-5p was identified as liver-specific miRNA (40–42). Several studies showed that expression levels of miR-122-5p can distinguish colorectal cancer patients from healthy controls and higher expression levels of miR-122-5p indicated the higher occurrence of metastasis in colorectal patients (43–46). Similarly, increased miR-122-5p expression levels in plasma (47) or serum EVs (48) were associated with the presence of distant metastases, worse survival and a higher risk of relapse (47). These findings are not in agreement with our results; however, several issues explain these inconsistencies. In the study of Maierthaler et al. (47) the cohort included only 25% of RC patients and the study of Sun et al. (48) did not specified the number of RC. Our study is exclusively focused on RC patients. Moreover, many studies were not focused on liquid biopsy and only analyzed expression in tumor tissues (or cancer cell lines). Similarly to us, the expression of serum miR-122-5p in patients with gastric cancer was significantly lower than in healthy controls and the authors also described correlation with the survival status – lower levels being associated with poor survival (49). Two other studies focused on plasma described a lower expression of miR-122-5p in gastric cancer patients than in healthy controls or patients with benign gastrointestinal diseases (50, 51). miR-122-5p plasma expression levels were also tested in patients with hepatocellular carcinoma (HCC). In the study of Amr et al. (52), authors showed that miR-122-5p was down-regulated in HCC patients compared to healthy controls and an analogous pattern was observed in the serum of HCC patients (53, 54). All these observations, describing down-regulation of miR-122-5p in serum and plasma of cancer patients, are consistent with our results.

To the best of our knowledge, the study of Kunigenas et al. is the only one focused on miR-142-5p in RC. The authors reported increased expression levels of this miRNA in rectal tissue compared to adjacent non-malignant mucosa (55). In colorectal cancer, the role of miR-142-5p seems to be more controversial. In two studies, miR-142-5p was described as up-regulated in colorectal tumor tissue compared to adjacent mucosa (56, 57). On the other hand, in the study of Kong et al., authors observed lower expression levels in tumor tissue (58). Unfortunately, these studies lack the stratification of patients according to the tumor localization and do not provide more detailed results about RC. Moreover, there is no study focusing on miR-142-5p in liquid biopsy.

The major advantage of our study is specific focus on RC patients. Moreover, all samples were enrolled in a single-center hospital and were of the same ethnicity and socio-cultural background. Plasma samples, as the source for miRNA expression analysis, were collected repeatedly within a year with the ability to monitor the therapy outcome. Generally, most studies are focused on the identification of diagnostic or prognostic biomarkers in body fluids collected only at the time of the diagnosis. The consecutive blood samplings as performed in the present study are usually lacking and this highlight the unique design of our study. In comparison with a traditional tissue biopsy, plasma samples bring advantages of easy, non-invasive collection that can be repeatedly collected overtime. The main limitation of this study is a relatively small cohort of patients with an available second sample and a lack of tissue material. The present study is, to our knowledge, the first study to evaluate changes in miRNA expression levels within the RC patients over the course of treatment, and their correlation with their follow-up.

## Data Availability Statement

The datasets presented in this study can be found in online repositories. The names of the repository/repositories and accession number(s) can be found below: NCBI Trace Archive NCBI Sequence Read archive https://www.ncbi.nlm.nih.gov/bioproject/727696.

## Ethics Statement

The studies involving human participants were reviewed and approved by Committee of the Institute of Experimental Medicine and the Thomayer Hospital, Prague. The patients/participants provided their written informed consent to participate in this study.

## Author Contributions

Design study and performance of discovery phase – VV, BP, and AN. Collection and preparation of samples – KC, ML, PS, and TB. Performance of validation phase – KC. Supervision *in vitro* analysis – AO. Statistical analysis – VN. Interpretation of data – BP, AN, and VV. Original draft preparation – KC. Writing – reviewing and editing – VV, BP, AN, PV, KC, TB, SV, ML, AO, PS, VN and JH. All authors contributed to the article and approved the submitted version.

## Funding

This project was supported by the Grant Agency of the Ministry of Health of the Czech Republic (AZV 17–30920A) and the Grant Agency of Charles University (GAUK 302119). The work was partially supported by LegaI taliana per La Lotta contro i Tumori (LILT) (to BP and AN) and by the Oncobiome European H2020 research project (grant number 825410 to AN).

## Conflict of Interest

TB received honoraria and research support from Roche, Bayer, Ipsen, Novartis, Merck, Bristol-Myers Squibb, and Servier (unrelated to the present article).

The remaining authors declare that the research was conducted in the absence of any commercial or financial relationships that could be construed as a potential conflict of interest.

## Publisher’s Note

All claims expressed in this article are solely those of the authors and do not necessarily represent those of their affiliated organizations, or those of the publisher, the editors and the reviewers. Any product that may be evaluated in this article, or claim that may be made by its manufacturer, is not guaranteed or endorsed by the publisher.

## Supplementary Material

Supplementary Figure 1 Expression analysis of miR-122-5p and miR-142-5p according to different stages of RC.

Supplementary Figure 2 Colony forming assay and cell cycle distribution figures for A) miR-122-5p and SW1463 cell lines, B) miR-122-5p and HRA16 cell lines, C) miR-142-5p and SW1463 cell lines, and D) miR-142-5p and HRA16 cell lines.

## Abbreviations

5-F, 5-fluorouracil; BP, biological processes; CC, cellular components; cDNA, complementary DNA; EVs, extracellular vesicles; GO, Gene Ontology; HCC, hepatocellular carcinoma; KEGG, Kyoto Encyclopedia of Genes and Genomes; MF, molecular functions; miRNAs, microRNAs; RC, rectal cancer; RT- qPCR, real-time quantitative polymerase chain reaction; TNM, tumor-node-metastasis.
