# Supplementary figures and images for: Analysis of MicroRNA Expression Changes During the Course of Therapy In Rectal Cancer Patients
Source: Front Oncol. 2021 Sep 2;11:702258. doi: 10.3389/fonc.2021.702258 (PMC8444897; doi:10.3389/fonc.2021.702258)

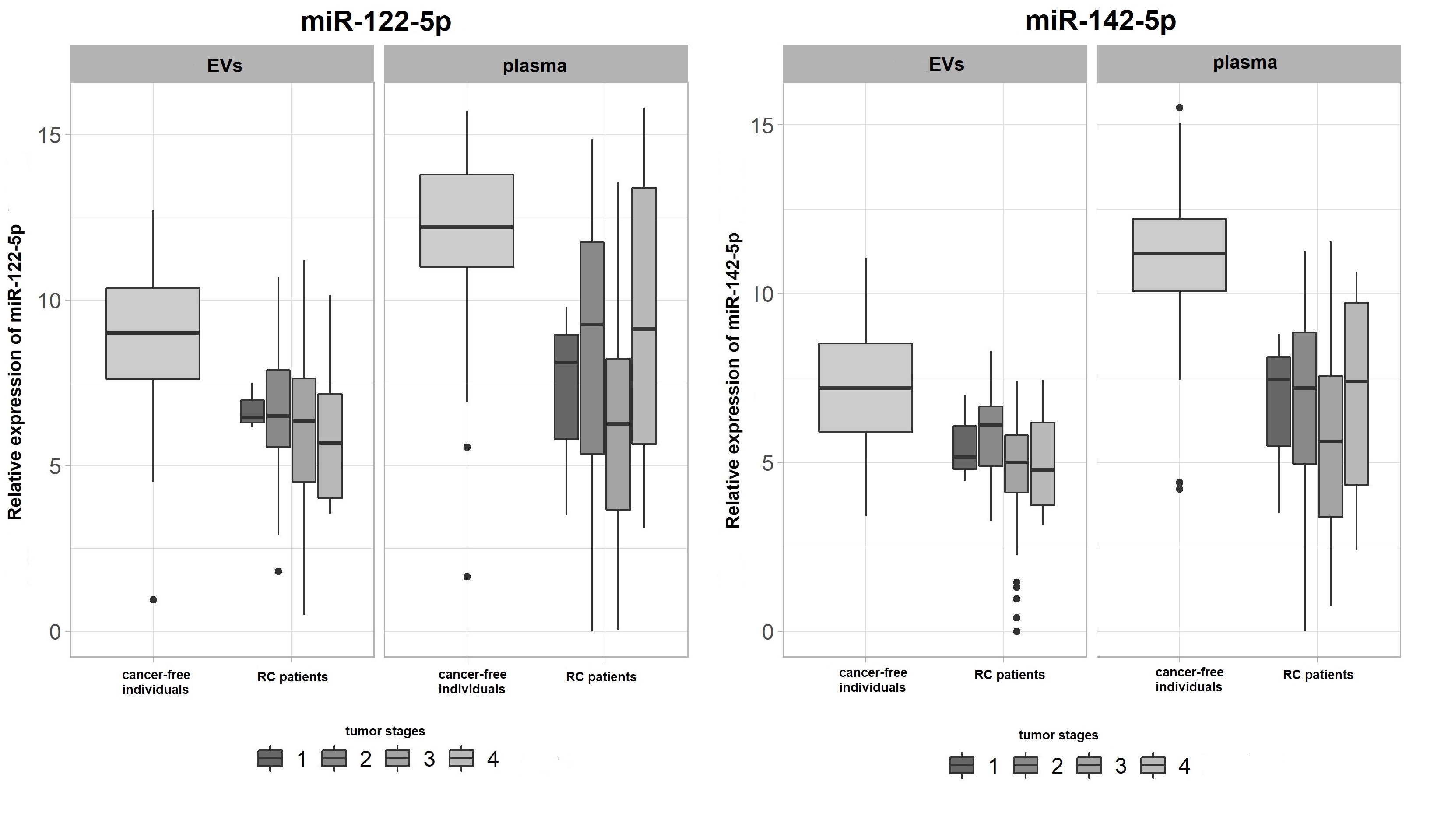

Supplement: Supplementary Figure 1 — Expression analysis of miR-122-5p and miR-142-5p according to different stages of RC. [file Image_1.jpeg]

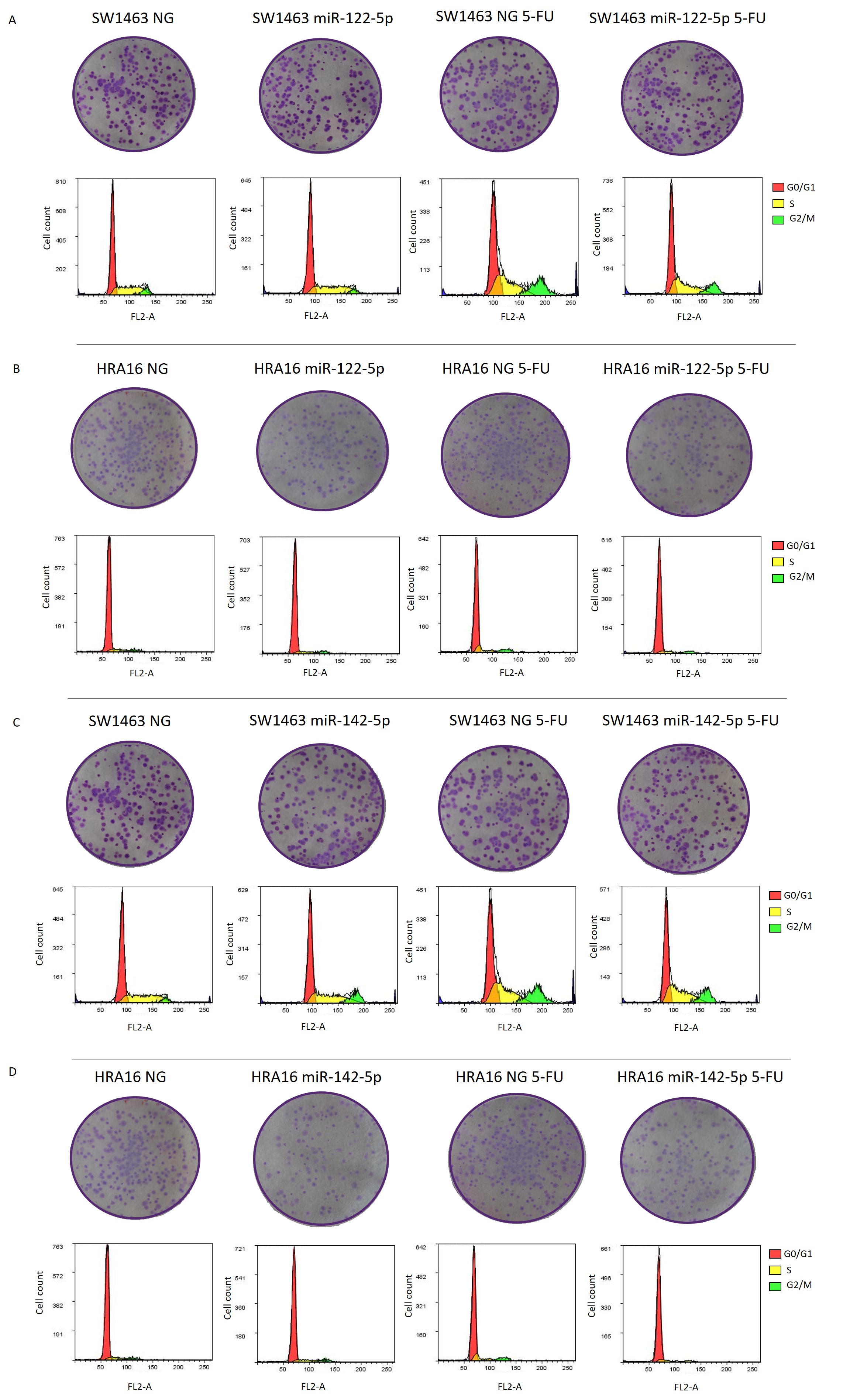

Supplement: Supplementary Figure 2 — Colony forming assay and cell cycle distribution figures for A) miR-122-5p and SW1463 cell lines, B) miR-122-5p and HRA16 cell lines, C) miR-142-5p and SW1463 cell lines, and D) miR-142-5p and HRA16 cell lines. [file Image_2.jpeg]
